# Fermented Wheat Powder Induces the Antioxidant and Detoxifying System in Primary Rat Hepatocytes

**DOI:** 10.1371/journal.pone.0083538

**Published:** 2013-12-31

**Authors:** Margherita La Marca, Pascale Beffy, Annalisa Pugliese, Vincenzo Longo

**Affiliations:** 1 Istituto di Biologia e Biotecnologia Agraria, CNR, Pisa, Italy; 2 Istituto di Fisiologia Clinica, CNR, Pisa, Italy; The Hong Kong Polytechnic University, Hong Kong

## Abstract

Many plants exhibit antioxidant properties which may be useful in the prevention of oxidative stress reactions, such as those mediated by the formation of free radical species in different pathological situations. In recent years a number of studies have shown that whole grain products in particular have strong antioxidant activity. Primary cultures of rat hepatocytes were used to investigate whether and how a fermented powder of wheat (Lisosan G) is able to modulate antioxidant and detoxifying enzymes, and whether or not it can activate Nrf2 transcription factor or inhibit NF-kB activation. All of the antioxidant and detoxifying enzymes studied were significantly up-regulated by 0.7 mg/ml Lisosan G treatment. In particular, NAD(P)H:quinone oxidoreductase and heme oxygenase-1 were induced, although to different degrees, at the transcriptional, protein and/or activity levels by the treatment. As for the Nrf2 transcription factor, a partial translocation of its protein from the cytosol to the nucleus after 1 h of Lisosan G treatment was revealed by immunoblotting. Lisosan G was also observed to decrease H_2_O_2_-induced toxicity

Taken together, these results show that this powder of wheat is an effective inducer of ARE/Nrf2-regulated antioxidant and detoxifying genes and has the potential to inhibit the translocation of NF-kB into the nucleus.

## Introduction

Humans are constantly exposed to factors causing oxidative stress including pollutants, radiation and oxidized food [1]. Oxidative stress, defined as a loss of balance between the cellular concentration of reactive oxygen species and the cell's antioxidant capacity, is implicated in the onset of various diseases [2]. The human body has several endogenous systems [3] with which it can protect itself against oxidative stress, but antioxidant factors acquired from food also play a key role. Indeed, certain micronutrients obtained from food have potent antioxidant properties and may play an important role in maintaining the oxidative/antioxidative balance, especially if the diet is rich in these constituents [4]. In recent years, a variety of vegetables that contain antioxidants potentially capable of preventing oxidative stress reactions, such as those mediated by the formation of free radical species have been studied. Tomatoes, spinach, green peppers and cabbage are important sources of vitamin C. *In vivo*, this vitamin acts as scavenger of oxygen radicals and also as competitive inhibitor of nitrosamine synthesis from nitrite and amines *in vivo* [5]. Isothiocyanates are a family of molecules which are abundant in cruciferous vegetables such as broccoli, watercress and cauliflower. Sulforaphane, the best known isothiocyanate, induces drug metabolizing enzymes such as glutathione S-transferase A1/2 isoforms and NAD(P)H:quinone oxidoreductase (NQO1) in primary hepatocytes [6]. Whole grains are good source of B group vitamins, vitamin E, some minerals (zinc, magnesium and phosphorous), and they contain a variety of phytochemicals such as phytoestrogens, phytate, proteins, polysaccharides, phenols and lignans that are able to minimize oxydive damage [7]. All these components may act synergically [8]. By contrast, refined grains have a reduced nutrient content as the milling process results in the loss of dietary fibre, vitamins, minerals, lignans, phytoestrogens, phenolic compounds and phytic acid [9]. Many wheat proteins contain reduced sulfhydryl groups, which can have some free radical scavenging activity. Phytic acid can protect tissues against oxidative reactions by sequestering and inactivating pro-oxidative transition metals [3]. In epidemiological studies, whole grain consumption is associated with improvements in body mass index (BMI) [10] and insulin sensitivity [11] as well as with lower incidences of type 2 diabetes [12], cardiovascular diseases [13], and colorectal cancer [14]. Little is known about how cereals effect cells and to our knowledge, no research has yet been done on the antioxidant properties of whole grain products in primary hepatocytes.

Several studies have shown that some phytochemicals can modulate antioxidant and phase II enzymes through the activation of nuclear factor E2-related protein (Nrf2) [15]. Nrf2 is a basic-leucine zipper transcription factor that under basal conditions, is present in an inactive form in the cytoplasm, bound to the Kelch-like ECH- associated protein 1 (Keap1) [16]. Various agents including Antioxidant Response Element (ROS) and weak electrophiles (e.g. isothiocyanates) can alter the Keap1-Nrf2 protein complex and free Nrf2 through phosphorilation or alkylation of one or more of the 27 cysteine residues in Keap1 [17]. When this occurs, Nrf2 translocates into the nucleus. Upon activation, Nrf2 dimerizes with a small Maf protein then binds to antioxidant responsive element (ARE) sites in the promoter regions of antioxidant and phase II genes, thereby inducing their transcription [18].

In recent years, many authors have suggested the existence of cross-talk between Nrf2/ARE and the nuclear factor-kappa B (NF-kB) signaling pathways in response to inflammation [19]–[21]. The Nrf2 and NF-kB signaling pathways interface at several points to control the transcription or function of downstream target proteins [22]. In addition, ROS now appear to act as second messengers in numerous signaling pathways [23]–[24]. One signaling pathway that engages in cross-talk with ROS involves NF-kB family transcription factors [25]–[27]. It had already been shown twenty years ago by Schreck and coworkers [28] that oxidative stresses, such as addition of extracellular hydrogen peroxide, can induced NF-kB nuclear translocation in several cell lines. The NF-kB family is made up of NF-kB1 (p50), NFkB2 (p52), RelA (p65), c-Rel and RelB. In the absence of stimuli, NF-kB, is associated with the inhibitor protein, IkBα, and sequestered in the cytosol. Upon stimulation with a NF-kB inducers, IkBα is rapidly phosphorylated on two serine residues (S32 and S36), which targets the inhibitor for ubiquitination and degradation by proteosome.

Lisosan G is a powder obtained from *Triticum Sativum* (wheat) and it is registered with the Italian Ministry of Health as a nutritional supplement. In the production process, the wholegrain is first ground to a rough powder. From this intermediary product, the bran and germ are separated and collected for further treatment which consists in the following: water is added to moisten the mix, then selected microbic starting cultures are inoculated to initiate fermentation. The starting cultures typically consist of a mix of lacto-bacillus and natural yeast strains. Once the product is sufficiently fermented, it is dried. The resulting dry powder is now Lisosan G, which is widely used in food production thanks to its rich nutritional content.

It contains vitamins, minerals and polyunsatured fatty acids as well as having significant antioxidant activity [29]. *In vivo*, Lisosan G protects against cisplatin induced toxicity [30], and a recent paper showed that Lisosan G helps prevent microcirculatory dysfunction [31]. The authors of these works suggested that the protective effect of Lisosan G could be associated with the attenuation of oxidative stress and the preservation of antioxidant enzymes. To date, no studies have attempted to identify the molecular mechanism that determines antioxidant properties of Lisosan G. For this reason, in the present study, we investigated the effects of Lisosan G on the antioxidant and drug-metabolising enzymes at transcriptional, catalytic and protein levels using cultures of primary rat hepatocytes.

## Materials and Methods

### 2.1 Chemicals

Lisosan G is registered as nutritional supplement by the Italian Minister of Health and was supplied by Agrisan Company, Larciano (PT), Italy. Collagenase; dexamethasone; insulin; glucagon; penicillin/streptomycin; ampicillin/kanamycin; fetal bovine serum; 4-(2-hydroxyethyl)-1-piperazineethanesulfonic acid (HEPES); Tween 20; phenylmethylsulfonyl fluoride (PMSF); leupeptin; apoprotein; pepstatin; tunicamycin; Williams E medium; bovine serum albumin (BSA); epidermal growth factor (EGF); β-nicotinamide adenine dinucleotide reduced (NADPH); ethylenediaminetetraacetic acid (EDTA); ethylene glycol tetraacetic acid (EGTA); glutathione (GSH); Glutathione disulfide (GSSG); and hydrogen peroxide (H_2_O_2_) were all supplied by Sigma-Aldrich (St. Louis, MO). Rabbit polyclonal anti-Nrf2 (sc-13032), anti-heme oxygenase-1 (sc-10789), NFkB (sc-7178), β-actin (sc-130657), PARP-1 (sc-25780) and goat anti-rabbit (1∶2000 or 1∶5000) were from Santa Cruz Biotechnology Inc. (Santa Cruz, CA). Collagen (type I) was prepared by the method of Beken et al. (1998).

### 2.2 Primary rat hepatocytes isolation, culture and treatments

Hepatocytes were isolated from 200–300 g Wistar male rats with free access to drinking water and food and on a 12 h light/dark cycle. The research with the use of animals was approved by the Italian Ministry of Health in compliance with European Community law n. 116/92. The approved protocol number is 10/09. The animals were anesthetized with an intraperitoneally injection of Zoletil ® (40 mg/kg) and then subjected to midline laparotomy in order to exteriorize the liver and isolate the portal vein. A needle was inserted into the portal vein and then the liver was perfused as described previously [32].

After filtration and centrifugation, the cell viability was determined by trypan blue exclusion. The cells were dispersed in Williams E medium containing 39 ng/ml dexamethasone, 0.5 U/ml insulin, 0.007 µg/ml glucagon, 5 µg/ml penicillin and streptomycin, 5 µg/ml ampicillin and kanamycin and 10% fetal bovine serum. The cells were plated at a density of 4.5×10^6^ cells/8 ml on 100 mm cell culture dish pre-coated with 3 ml of collagen (type I) solution (1 mg/ml). The cultures were maintained at 37°C in 5% CO_2_ in a humidifier incubator. After 5 h, the medium was replaced with serum-free Williams E medium supplemented with 2% BSA, 7.5 µg/ml hydrocortisone 21-hemisuccinate sodium salt and EGF 20 ng/ml. Cultures were maintained in this medium at 37°C and 5% CO_2_ for 24 h. After this period, the medium was removed and a second layer of type I collagen was added to create a collagen-gel sandwich culture [33] and after 45 minutes serum-free Williams E medium was added again. The cells were maintained for additional 24 h before treatments. Cells treatments were divided in four different groups: in the first group (control), the cells were treated with medium only; in the second one with Lisosan G 0.7 mg/ml (Lis); in the third group with H_2_O_2_ 200 µM and in the last group, the H_2_O_2_ 200 µM was added after 1 h pre-treatment with Lisosan G 0.7 mg/ml (Lis+H_2_O_2_). We have used different concentrations of H_2_O_2_ (10–500 µM) and we chose 200 µM (good toxicity); we also used for Lisosan G different concentrations from 0.1 to 2.8 mg/ml and 0.7 mg/ml was the best concentration in terms of maximum protective effect and without toxicity. The cytotoxic effects compared with the vehicle-only controls, was also measured by lactate dehydrogenase assay (data not shown). We have also performed experiments in function of time of treatments of H_2_O_2_ and Lisosan G and we chose the time of treatment on the basis of the best results.

### 2.3 Enzymatic activities

After 24 h the end of treatment, the medium was removed and a collagenase solution was added. After 30 minutes, recovered cells were centrifuged (400× g) for 3 minutes at 4°C. The cell pellet was sonicated and used for the microsomal preparation [34]. Total protein concentration was determined by the method of Lowry [35]. NAD(P)H:quinone oxidoreductase (NQO1) activity was measured by the method of Bensen et al. [36]. Glutathione-S-transferase (GST) activity was quantified as previously described by Habig et al. [37] using 1-chloro-2,4-dinitrobenzene as substrate. Catalase activity was monitored following the H_2_O_2_ decomposition at 240 nm, as described by Cao and Li [38]. Heme oxygenase-1 (HO-1) activity was determined by the method of Naughton et al. [39]. Lactate dehydrogenase activity was assayed as previously described [40]. Reduced GSH was measured using the method previously described by Hissin and Hilf [41]. Lipid peroxidation was monitored by determining the production of malondialdehyde (MDA)-like products, quantified by the reaction with thiobarbituric acid (TBA) as reported by Stacey et al. [42].

### 2.4 RNA Extraction and cDNA synthesis

Total cellular RNA was extracted from primary rat hepatocytes 4 h after ITC treatment, using the RNeasy Mini Kit (Qiagen, Valencia, CA), following the supplied protocol. RNA was quantified using NanoDrop (Celbio, Mi, Italy); its purity and integrity were evaluated by checking the absorbance ratio at 260–280 nm and assessing the sharpness of 18S and 28S ribosomal RNA bands on agarose gel stained with ethidium bromide. Genomic DNA elimination and reverse transcription of total RNA were performed using QuantiTect Reverse Transcription Kit (Qiagen).

### 2.5 RT-PCR

Two microliters of cDNA were added to a PCR Master Mix (GoTaq Green Master Mix, Promega, Madison, WI) for the amplification reaction (various cycles) performed using for each transcript 400 nM of forward–reverse primers for heme oxygenase-1 (GenBank accession no. NM_012580.2), NQO1 (GenBank accession no. NM_017000.3), β-actin, as housekeeping gene, (GenBank accession no. NM_031144.2) and the annealing temperature indicated in Table 1. The DNA fragments were separated on ethidium bromide-stained 1% agarose gel and visualized by transillumination with ultraviolet light. Bands obtained from five independent rat experiments were quantified by an Image J software. The results have been normalized to β-actin levels and are expressed as percentages of control. Results are reported as means ± SD of cells from five independent experiments using five rats.

**Table 1:** Primer pairs, annealing temperature and product size for RT-PCR experiments.

Gene	Forward (5′-3′)	Reverse (5′-3′)	Annealing T. (°C)	Product size (bp)
NQO1	ACTCGGAGAACTTTCAGTACC	TTGGAGCAAAGTAGACTGGT	59	492
HO-1	CAGGGTGACAGAAGAGGCTAAGAC	TGAGGACCCATCGCAGGAG	66	229
β-actin	CCCCATTGAACACGGATT	CATCTTTTCACGGTTGGCCTTA	67	150

### 2.6 Preparation of nuclear fractions

Nuclear and cytosolic extracts were prepared by previously established methods [43]. Briefly, hepatocytes were washed twice with 1× phosphate buffer saline (PBS). Cells were then harvested in 1 ml of PBS and centrifuged at 800 g for 3 min at 4°C. The pellet was carefully resuspended in 200 µl of cold hypotonic buffer, consisting of 10 mM HEPES (pH 7.9), 10 mM KCl, 0.1 mM EDTA, 0.1 mM EGTA, 1 µM dithiothreitol and complete protease inhibitor cocktail (Sigma antiprotease cocktail P8340). After addition of NP40 to a final concentration of 0.3%, the cells were vortexed and centrifuged at 800 g for 3 min at 4°C. The resulting nuclear pellet was resuspended in 30 µl of cold nuclear extraction buffer (20 mM HEPES (pH 7.9), 0.4 M NaCl, 1 mM EDTA, 1 mM EGTA, 1 µM dithiothreitol, 25% glycerol and protease inhibitors) and incubated on ice for 30 min. The nuclear extract was finally centrifuged at 15000 g for 15 min at 4°C. The supernatant containing nuclei proteins was aliquoted and stored at −30°C.

### 2.7 Immunoblot analysis

Nuclear and microsomal proteins from primary rat hepatocytes were separated according to Laemmli [44] on SDS-10% (v/v) 1.0 mm thick polyacrylamide gels and then electrophoretically transferred onto nitrocellulose membranes following the method of Towbin et al. [45]. Antibodies used were anti-Nrf2 (1: 1000, sc13032, Santa Cruz Biotechnology, Heidelberg, Germany), anti heme oxygenase-1 (1∶1000, sc-10789, Santa Cruz Biotechnology, Heidelberg, Germany), β-actin (1∶1000, sc-130657, Santa Cruz Biotecnology, Heidelberg, Germany), PARP-1 (1∶1000, sc-25780, Santa Cruz Biotecnology, Heidelberg, Germany), NF-kB (1∶1000, sc-7178, Santa Cruz Biotecnology, Heidelberg, Germany) and goat anti-rabbit (1∶2000 or 1∶5000). Immunoreactive proteins were visualized with a chemiluminescence reaction kit (EuroClone, Mi, Italy) and bands obtained from five independent rat experiments were electronically scanned and quantified by an Image J software.

### 2.8 Statistical analysis

Results are reported as means ± SD. Statistical significance was determined by Student's t-test for comparison between control and treated groups or the one-way ANOVA and the Dunnet tests. The statistical program used was Graphpad Prism 4. P value<0.05 was considered to be significant.

## Results

### 3.1 Effect of Lisosan G on antioxidant and phase II enzymes

**Figure 1:**
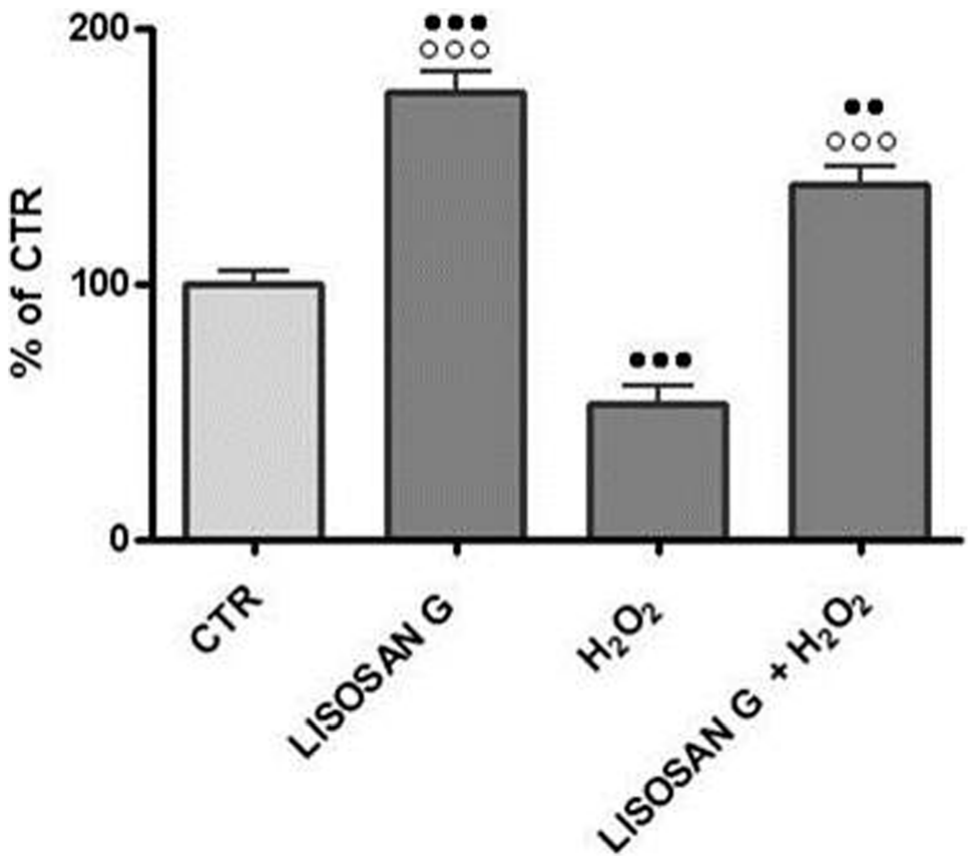
Effect of Lisosan G on GSH levels. The activity was measured in homogenates of 24 h treated hepatocytes. Results are expressed as percentages of control activity (125.67±28.04 nmol/mg prot.). Mean ± SE of cells from five rats. ••Significantly different from control, p<0.01. ••• p<0.001. ○○○ Significantly different from H_2_O_2_, p<0.001.

To verify the ability of Lisosan G to protect hepatocytes from damage caused by oxidative stress, we treated a first group of cells with hydrogen peroxide, while a second group was pretreated with Lisosan G prior to exposure to hydrogen peroxide. As shown in fig. 1, 200 µM H_2_O_2_ caused a decrease in reduced glutathione levels, whereas pretreatment with 0.7 mg/ml Lisosan G followed by H_2_O_2_, raised the amount of intracellular GSH to levels above control cells values. Lisosan G alone was able to increase reduced glutathione levels in hepatocytes treated for 24 h.

**Figure 2:**
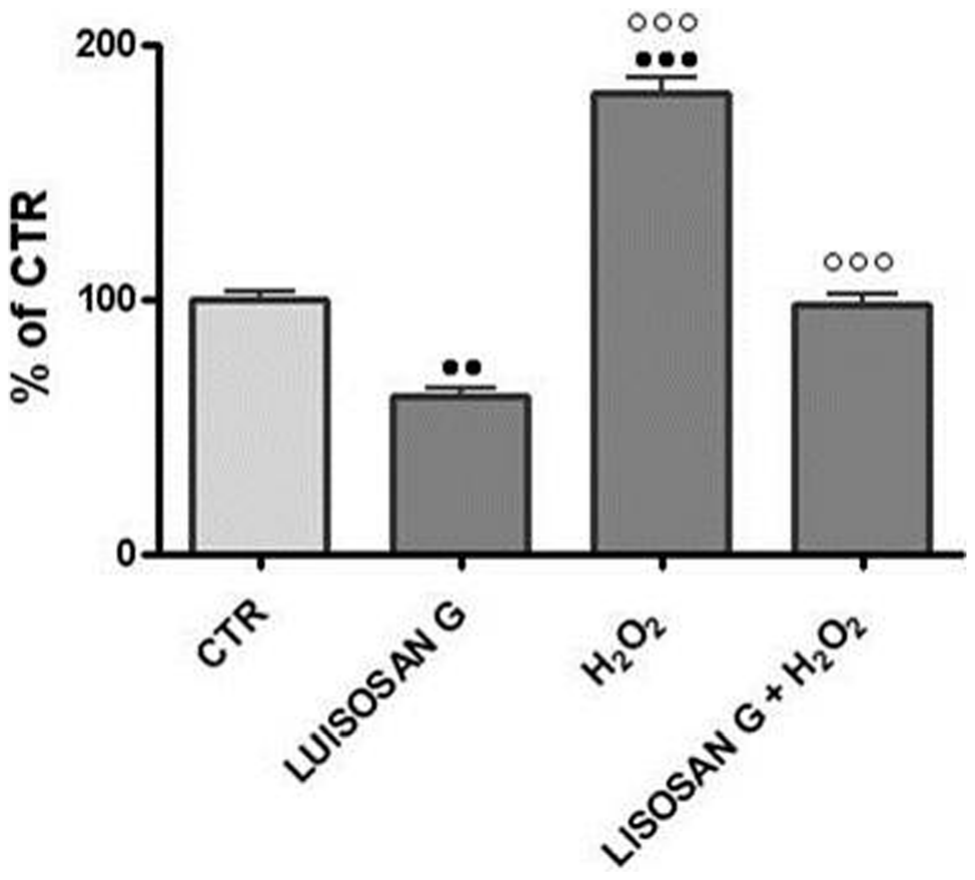
Effect of Lisosan G on lipid peroxidation. The activity was measured in microsomes from 24 h treated hepatocytes. Results are expressed as percentages of control activity (32.82±2.07 mUA/mg prot.). Mean ± SE of cells from five rats. ••Significantly different from control, p<0.01. ••• p<0.001. ○○○Significantly different from H_2_O_2_, p<0.001.

As a biomarker for lipid peroxidation, the concentration of malondialdehyde (MDA) in microsomes was measured in cells treated with H_2_O_2_ which had been pretreated for 1 h with Lisosan G. Fig. 2 shows the effect of preincubation with Lisosan G on H_2_O_2_-induced lipid peroxidation. Exposure of cells to H_2_O_2_ for 24 h significantly increased lipid peroxidation while preincubation of cells for 1 h prevented this from occurring. Also, Lisosan G on its own reduced lipid peroxidation levels compared to control (CTR) cells.

**Figure 3:**
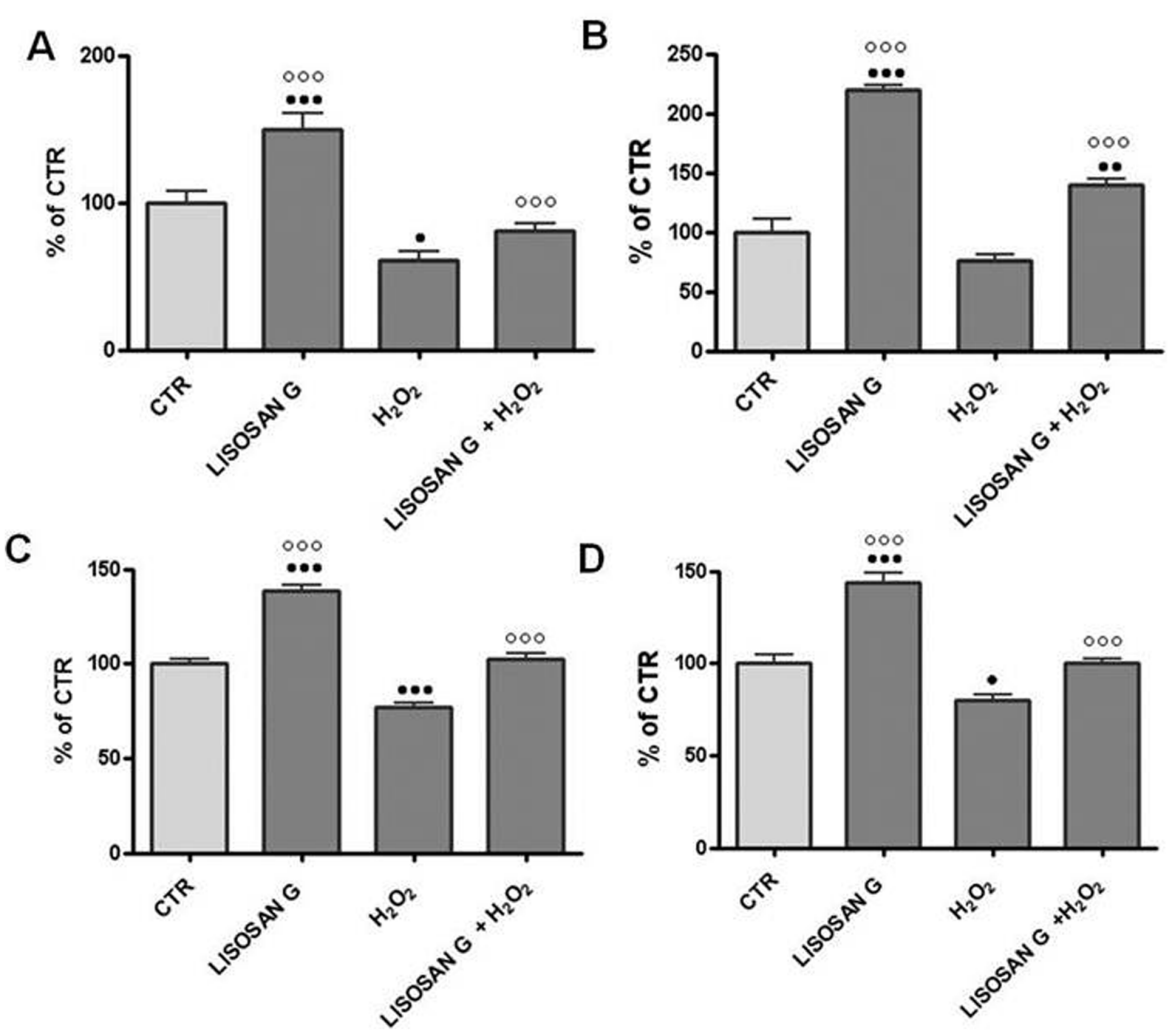
Effect of Lisosan G on activity of antioxidant and phase II drug-metabolizing enzymes. NAD(P)H:quinone oxidoreductase (control value: 53.23±2.5 nmol/min/mg prot) (A); Heme oxygenase-1 (control value: 9.57±3.86 pmol/min/mg prot) (B); Glutathione-S-transferase (control value: 239.5±12.12 nmol/min/mg prot) (C); Catalase (control value: 147.41±11.1 nmol/min/mg prot) (D). Activities were measured in microsomes or cytosol of control cells (CTR) and 24 h treated cells. Results are expressed as percentages of control values. Mean ± SE of cells from five rats. • Significantly different from controls, p<0,05. ••p<0.01. ••• p<0.001. ○○○ Significantly different from H_2_O_2_, p<0.001.

In the next set of experiments, the effect of Lisosan G on antioxidant and phase II enzyme activity was evaluated in microsomes and the cytosol of control hepatocytes and hepatocytes treated for 24 h. The enzymes chosen were NQO1, HO-1, glutathione-S-transferase (GST) and catalase. H_2_O_2_ treatment reduced NQO1 activity, but NQO1 activity remained high when a 1 hour pre-treatment with (fig. 3A) Lisosan G preceeded the H_2_O_2_ treatment. It is interesting to note that Lisosan G alone caused an increase in NQO1 activity (about 1.5 fold of control value). H_2_O_2_ also caused a decrease in HO-1 activity compared to control cells, but pretreatment with Lisosan G before H_2_O_2_ treatment elevated the activity above the level of CTR. HO-1 activity was significantly induced by Lisosan G treatment (about 2.2 fold of control value) (fig. 3B). As for GST, its activity decreased after the 24 h treatment with H_2_O_2_, but pretreatment with Lisosan G restored it nearly to control values (fig. 3C). This phase II activity was induced to about 1.2 times the control value by the Lisosan G treatment. We observed a 1,4 fold increase in catalase activity (fig. 3D), following treatment with Lisosan G and a significant decrease after the 24 h H_2_O_2_ treatment. However, the pretreatment with Lisosan G before exposure to H_2_O_2_, restored its activity to the same level of the CTR.

**Figure 4:**
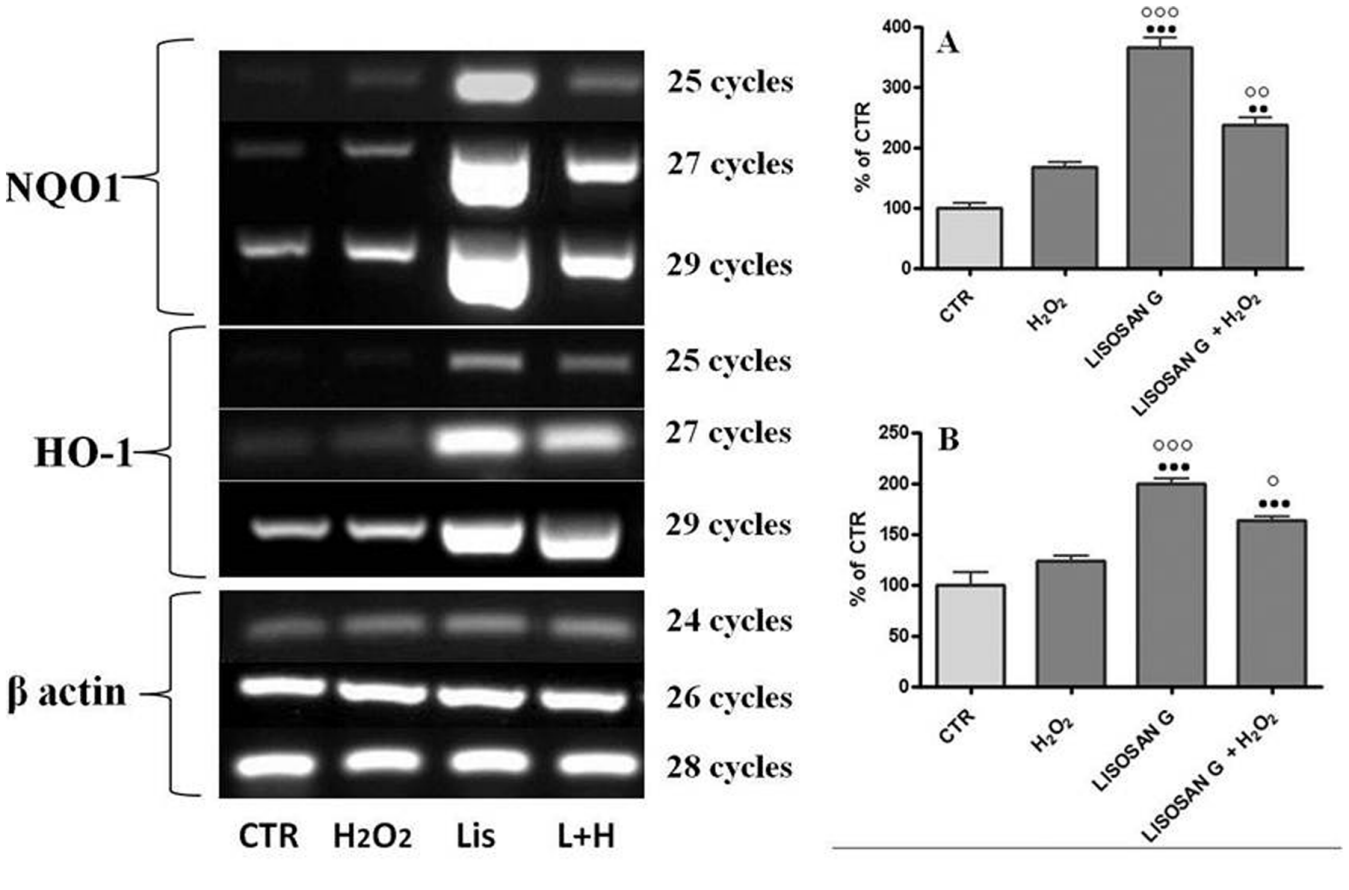
A representative RT-PCR analysis. NQO1 (A), HO-1 (B) genes performed with 25, 27 and 29 cycles in primary rat hepatocytes of control (CTR) and treated with Lisosan G or Lisosan G+H_2_O_2_. PCR products were separated by electrophoresis on agarose gels and stained with ethidium bromide. Quantitative representation of the RT-PCR analysis is reported in the histograms and the results have been normalized to β-actin levels and are expressed as percentages of control. Mean ± SE of cells from five independent experiments using five rats. •• Significantly different from controls, p<0.01. ••• p<0.001. ○ Significantly different from H_2_O_2_, p<0.05. ○○○ p<0.001.

NQO1 and HO-1 were chosen to analysis of the response to Lisosan G at the transcriptional level. The expression of their transcripts in primary rat hepatocytes were analyzed by semi-quantitative RT-PCR, using the sets of primers listed in Table 1. Cells were treated with 0.7 mg/ml Lisosan G and 200 µM H_2_O_2_ for 4 h. As shown in fig. 4A, H_2_O_2_ didn't cause any significant change in NQO1 expression, but both the treatment with Lisosan G only and Lis+H_2_O_2_ increased its expression compared to the control levels.

A similar trend was found for HO-1 expression, although to different extents (Fig. 4B). For this gene, both Lisosan G alone and Lisosan G followed by H_2_O_2_ caused a rise in HO-1 expression but in this case, H_2_O_2_ alone also induced the gene.

**Figure 5:**
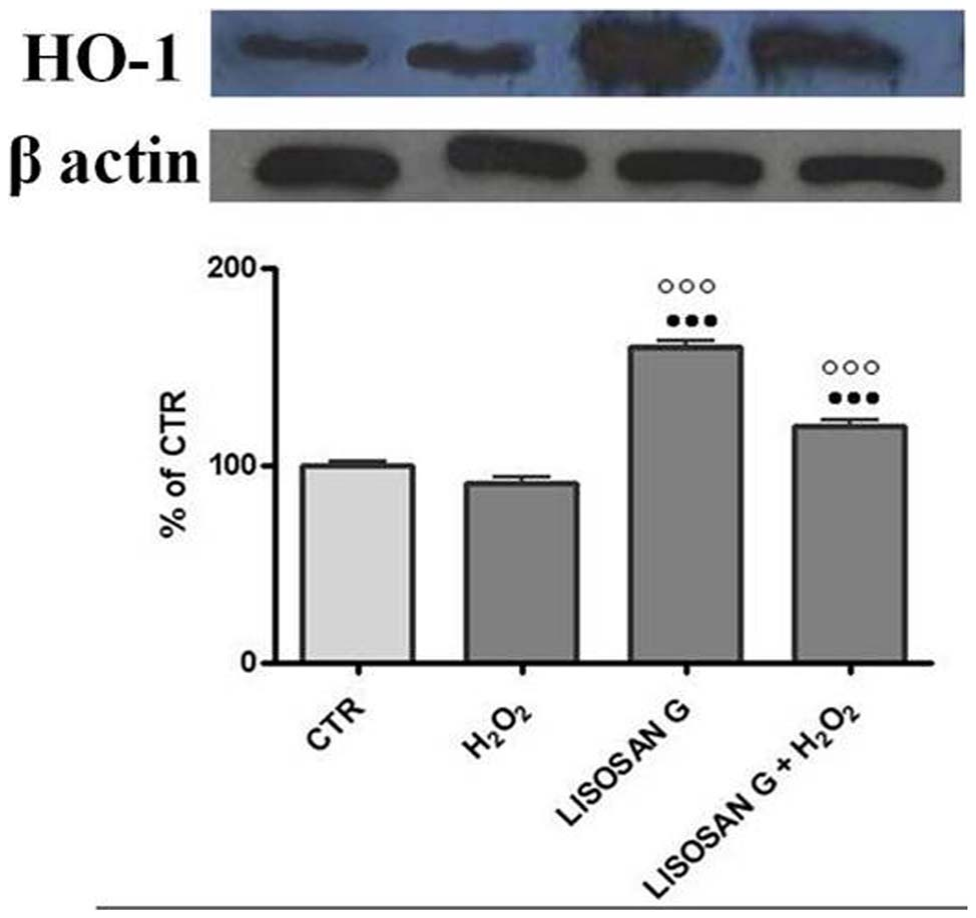
Western blot analysis heme oxygenase-1 protein. In microsomes (50 µg) of control (CTR) cells and cells treated for 24 h with Lisosan G or Lisosan G+H_2_O_2_. Microsomal samples were subjected to SDS-PAGE, electrophoretically transferred to a nitrocellulose membrane, and probed with polyclonal antibodies raised against rat HO-1. Densitometric analysis of the western blot data are shown in the histogram. The results have been normalized to β-actin levels and are expressed as percentages of control. Mean ± SE of cells from five independent experiments using five rats. ••• Significantly different from controls, p<0.001. ○○○ Significantly different from H_2_O_2_, p<0.001.

The effect of Lisosan G on heme oxygenase-1 was also assessed at the protein level by western blot (fig. 5). In microsomes from all hepatocytes treated with H_2_O_2_ no effect was noticed after 24 h treatment. On the contrary, the pre-treatment with Lisosan G before H_2_O_2_, raised the HO-1 protein level 1.6 fold above the CTR value, in agreement with results from the activity assays and transcription analysis.

### 3.2 Effect of Lisosan G on Nrf2 and NF-kB

**Figure 6:**
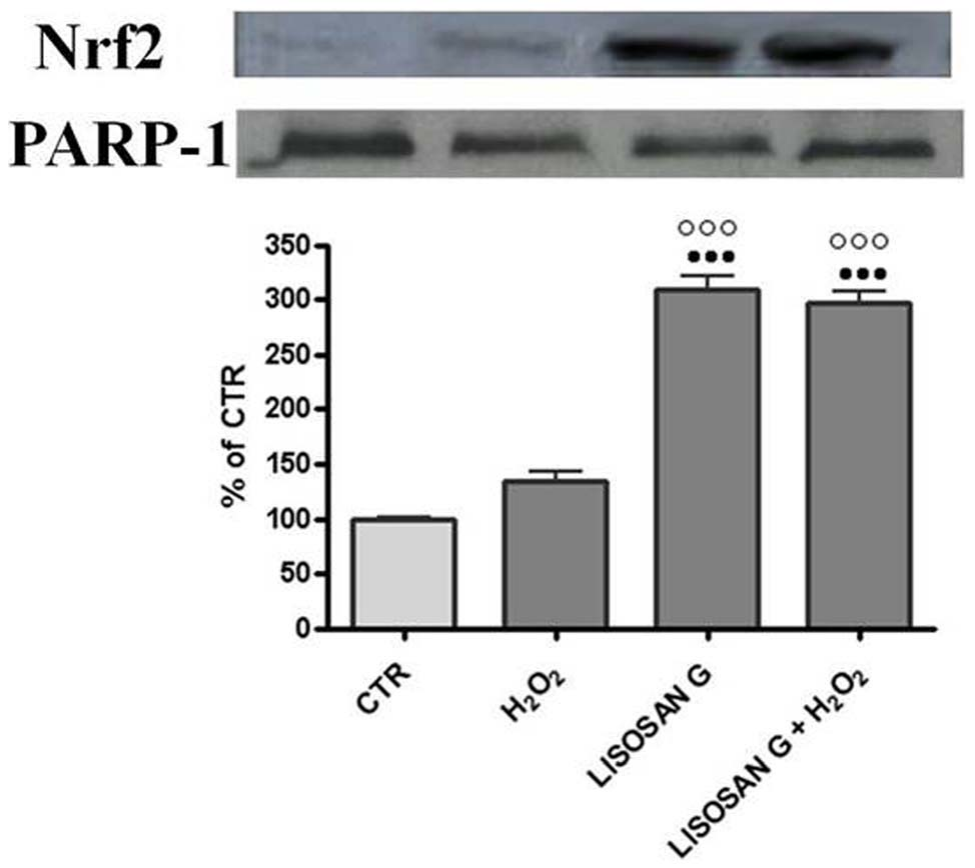
Western Blotting analysis of Nrf2 in nuclear extracts. Control cells (CTR) and cells treated with Lisosan G or Lisosan G+H_2_O_2_. Protein samples (30 µg) were subjected to SDS-PAGE, electrophoretically transferred to a nitrocellulose membrane, and probed with polyclonal antibodies raised against rat Nrf2. Densitometric analysis of the western blot data are shown in the histogram. The results have been normalized to PARP-1 levels and are expressed as percentages of control. Mean ± SE of cells from five independent experiments using five rats. ••• Significantly different from controls, p<0.001. ○○○ Significantly different from H_2_O_2_, p<0.001.

Since a major component of cellular defense against oxidative or electrophilic stress is the activation of the Nrf2/ARE signaling pathway [46], we verified whether Lisosan G was able to activate this important transcription factor in primary rat hepatocytes after 1 h of treatment. We analyzed by western blot nuclear fractions prepared from control and treated-cells (fig. 6). The protein band was faint in control and H_2_O_2_ nuclei. On the contrary, Nrf2 was clearly visible in the nuclear extracts of hepatocytes treated with Lisosan G and with Lisosan G+H_2_O_2_. The results have been normalized to PARP-1 levels.

**Figure 7:**
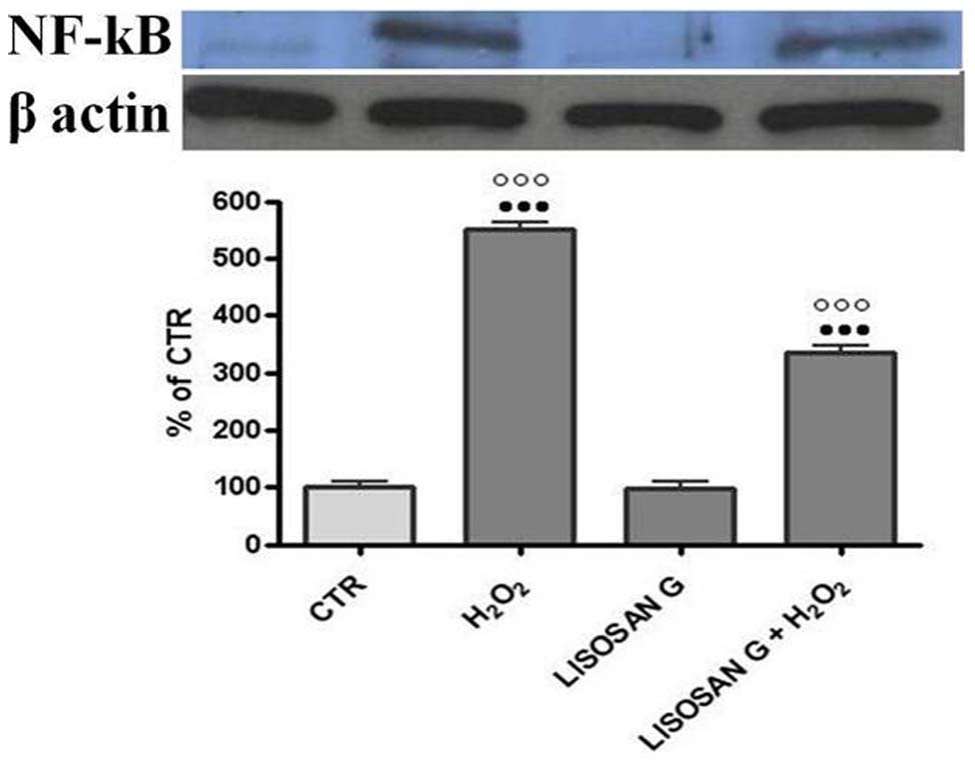
Western Blotting analysis of NF-kB. In nuclear extracts of control cells (CTR) and cells treated with Lisosan G or Lisosan G+H_2_O_2_. Protein samples (30 µg) were subjected to SDS-PAGE, electrophoretically transferred to a nitrocellulose membrane, and probed with polyclonal antibodies raised against rat NF-kB. Densitometric analysis of the western blot data are shown in the histogram. The results have been normalized to β-actin levels and are expressed as percentages of control. Mean ± SE of cells from five independent experiments using five rats. ••• Significantly different from controls, p<0.001. ○○○ Significantly different from H_2_O_2_, p<0.001.

As cross talk between Nrf2 and NF-kB is an area of intense interest, we investigated whether Lisosan G treatment would prevent the NF-kB translocation to the nucleus, caused by hydrogen peroxide. We analyzed the nuclear fraction by western blot (fig. 7). NF-kB was distinctly induced in H_2_O_2_ treated hepatocytes. The signal in control cells was similar to that of cells treated with Lisosan G, both very weak. The signal from nuclear extracts of hepatocytes treated with Lisosan G+H_2_O_2_ is fainter than that obtained in hydrogen peroxide treated cells. The results have been normalized to β-actin levels.

## Discussion

In the present study, we investigated whether a fermentated wheat powder, Lisosan G, was able to protect against H_2_O_2_ induced oxidative stress and whether it was able to modulate phase 2 enzymes by activating the Nrf2 protein and causing its translocation into the nucleus. We also analyzed its ability to prevent NF-kB nuclear translocation. We used sandwich cultures of primary rat hepatocytes, a unique *in vitro* system that preserves hepatic cytomorphology, as well as its drug metabolism, deposition and toxicity, to allow close resemblance with *in vivo* parameters [47]. When cultured between two layers of gelled collagen, hepatocytes also retain their ability to form intact canalicular networks and they retain their polarized excretory function [48].

Lisosan G defended cells against damage induced by H_2_O_2,_ and increased NQO1, HO-1, GST and catalase activity, suggesting that Lisosan G has antioxidant properties and confirming earlier *in vivo* studies which had demonstrated the ability of Lisosan G to elevate GST, NQO1, catalase and GSH peroxidase activity [29], [30]. The induction of HO-1 represents an important event in adaptive cellular response to different oxidative stimuli [20]. Indeed, we detected an increase in HO-1 at the catalytic, transcriptional and protein level, both in response to Lisosan G treatment alone and to Lisosan G pretreatment followed by H_2_O_2_ induced oxidative stress. Several classes of phytochemicals such as phenols, flavonoids, isothiocyanates, organosulfurs, and indoles can induce detoxifying enzymes such as NQO1 and HO-1 [49]. Curcumin, for example, was observed to exert hepatoprotective properties against ethanol-induced oxidative stress, via dose- and time-dependent induction of HO-1, in primary rat hepatocytes [50]. We noticed that Lisosan G increased GSH levels both when used on its own and as pre-treatment before H_2_O_2_. GSH is a multifunctional intracellular non-enzymatic antioxidant and it is considered to be the major thiol-disulphide redox buffer of the cell [51]. The protective role of GSH against oxidative stress depends on the equilibrium between thiol reduced (GSH) and disulfide-oxidized forms [52]. Cellular GSH depletion has been found to be associated with decreased cell proliferation in vascular endothelial cells [53]. Since the level of GSH is an important factor in the protection of cells, we believe that Lisosan G has excellent cytoprotective ability.

NQO1, HO-1 and GST gene expression is regulated by several key transcriptional factors located in the upstream region [20]. We found that in rat hepatocytes the induction of these enzymes occurred through the activation of the Nrf2 protein and its subsequent translocation into the nucleus. This mechanism has also been observed in hepatic cell lines in previous studies [54]. In this paper, we have shown by immunoblotting that this fermented wheat powder was able to activate Nrf2 after just 1 h of treatment.

It is important to note that the Nrf2 and NF-kB signaling pathways interface at several points to control the transcription or function of downstream target proteins. We therefore tried to understand if Lisosan G was able to regulate NF-kB. It is known that released NF-kB translocates into the nucleus where it regulates the transcription of genes for chemokines, cytokines, immunoreceptors, cell-adhesion molecules, growth factors, tumor necrosis factor α (TNFα), inducible NOS (iNOS), interleukin-1 (IL-1), interleukin-6 (IL-6) and cyclooxygenase (COX-2) [55]. Some phytochemicals can prevent the activation of NF-kB. Sulforaphane, for exemple, reduces the DNA binding of NF-kB in Raw 264.7 macrophages without affecting IkB [56]. Several chemopreventive agents trigger Nrf2 signaling with a concomitant repression of NF-kB and its target genes [22]. Chalcone (a flavonoid) has been shown to induce Nrf2 while inhibiting the activation of NF-kB in endothelial cells [57]. 3H-1,2-Dithiole-3-thione reduces the nuclear translocation and DNA binding of NF-kB, and also induces changes in phosphorilation of IkB in rat hepatocytes [58]. Our results show that the hydrogen peroxide treatment increases the amount of NF-kB protein in nucleus and the pretreatment with Lisosan G decreased it.

The ability of Lisosan G to induce phase II enzymes via Nrf2 and inhibit NF-kB activation, may be linked to its composition [29]. Lisosan G contains has linoleic (20: 4n-6) and linolenic (18: 3n-3) acids, which are defined “essential” fatty acids since they are not synthesized in the human body and are mostly obtained from diet [59]. They appear to play an important role in the prevention and treatment of a number of diseases (coronary disease, arthritis, inflammatory disorders) [60]. Pal and Ghosh [61] have shown that activity of antioxidant enzymes such as catalase, superoxide dismutase, glutathione peroxidase in liver and kidney decrease significantly in response to oxidative stress generated by methylmercury (MeHg); but histopathology of liver and kidney cells showed that administration of α-linolenic acid restored, in rat, all the altered parameters and also reduced lipid peroxidation. A recent paper showed a consistently presence of flavonoids and phenolic components in Lisosan G [62].

Lisosan G also contains minerals such as iron, zinc, copper and it is rich in vitamins (B1, B2, B6 and E). Vitamin E is an important natural antioxidant, and its most common and biologically active form is α-tocopherol. Unpublished results have shown that α-lipoic acid (ALA) is among the various components in Lisosan G, and that it is present in a concentration of 66 mg/kg.

ALA is a thiol antioxidant found in vegetables, including broccoli, spinach and tomatoes [63]. In human leukemia HL-60 cells and neuroblastoma SH-SY5Y cells, ALA upregulates NQO1 gene transcription [64], [65]. In addition, ALA, as sulforaphane, increases phase II protein levels in Clone 9 cells [66]. Ogborne et al. [67], have shown that in human monocytic cells, ALA induces HO-1 expression via Nrf2. It is interesting to note that ALA is an inhibitor of NF-kB [63] and it inhibits the NF-kB-dependent expression of metalloproteinase-9 *in vitro* [68]. We can suppose that components of Lisosan G, such as linoleic and linolenic acids, α-lipoic acid, flavonoids and phenols, which are known to cross the membrane, could be responsible of activation of NRF2 and of the inhibition of NF-KB in the primary hepatocyte cells.

This study has established for the first time in primary rat hepatocytes that Lisosan G can modulate phase 2 enzymes through the activation of Nrf2 pathway. We have demonstrated the Lisosan G decreases the H_2_O_2_-induced translocation of NF-kB to the nucleus. It seems likely that the beneficial effects of Lisosan G derive from its high phytochemical and vitamin content.
